# Strictures upon the Popular Prejudices in Regard to the Treatment of Measles

**Published:** 1852-09

**Authors:** A. W. Benton

**Affiliations:** Sterling


ART. V.—*Strictures upon the Popular Prejudices in regard to the Treatment of Measles.* By A. W. BENTON, M.D.

THE measles were quite prevalent in this community the past spring, assuming a mildly inflammatory type, and requiring but little medical treatment, with the exception of a few cases which were engrafted on unsound lungs, or rendered dangerous by exposure or injudicious treatment.

I never before so fully appreciated the strong and peremptory language of Dr. Dewees in proscribing "hot teas and stimulating drinks" in this disease. It seemed to be, with a few exceptions, the universal opinion of the people in this vicinity that the measles must be driven out with hot teas and whiskey punch. Accordingly they put it in practice, and if the patient grew worse it was a sure indication to them that the stimulants must be increased; "the measles must be driven out or the patient would die." It never occurred to their minds that it was the stimulants, and not the measles, that made them worse. In many cases a high grade of fever and a threatening pulmonary inflammation was excited, which the unaided powers of nature could scarcely overcome.

But what is most to be deplored is that some medical men have countenanced this treatment, and put it in practice, with the slight alteration of *brandy sling* for "whiskey punch;" whether from a conviction of its propriety, or a desire to gain popularity by pandering to the ignorance and superstition of the people, I cannot say.

I am more and more convinced every year, that the dignity of the medical profession can never be maintained as it should be, until a knowledge of the sciences, more immediately connected with the healing art, is more generally diffused among the common people. So long as ignorance of anatomy, physiology and therapeutics prevails, quackery will ride triumphant over skill and science.

STERLING, August 7, 1852.

